# Simulation-based estimation of mean and standard deviation for meta-analysis via Approximate Bayesian Computation (ABC)

**DOI:** 10.1186/s12874-015-0055-5

**Published:** 2015-08-12

**Authors:** Deukwoo Kwon, Isildinha M. Reis

**Affiliations:** Sylvester Comprehensive Cancer Center, University of Miami, Miami, FL 33136 USA; Department of Public Health Sciences, University of Miami, Miami, FL 33136 USA

**Keywords:** Meta-analysis, Sample mean, Sample standard deviation, Approximate Bayesian Computation (ABC)

## Abstract

**Background:**

When conducting a meta-analysis of a continuous outcome, estimated means and standard deviations from the selected studies are required in order to obtain an overall estimate of the mean effect and its confidence interval. If these quantities are not directly reported in the publications, they must be estimated from other reported summary statistics, such as the median, the minimum, the maximum, and quartiles.

**Methods:**

We propose a simulation-based estimation approach using the Approximate Bayesian Computation (ABC) technique for estimating mean and standard deviation based on various sets of summary statistics found in published studies. We conduct a simulation study to compare the proposed ABC method with the existing methods of Hozo et al. (2005), Bland (2015), and Wan et al. (2014).

**Results:**

In the estimation of the standard deviation, our ABC method performs better than the other methods when data are generated from skewed or heavy-tailed distributions. The corresponding average relative error (ARE) approaches zero as sample size increases. In data generated from the normal distribution, our ABC performs well. However, the Wan et al. method is best for estimating standard deviation under normal distribution. In the estimation of the mean, our ABC method is best regardless of assumed distribution.

**Conclusion:**

ABC is a flexible method for estimating the study-specific mean and standard deviation for meta-analysis, especially with underlying skewed or heavy-tailed distributions. The ABC method can be applied using other reported summary statistics such as the posterior mean and 95 % credible interval when Bayesian analysis has been employed.

**Electronic supplementary material:**

The online version of this article (doi:10.1186/s12874-015-0055-5) contains supplementary material, which is available to authorized users.

## Background

In medical research, it is common to conduct a systematic review and meta-analysis to provide an overall estimate of a clinical treatment outcome from a set of individual studies. When the outcome is continuous, in order to conduct meta-analysis, we need estimated means and the corresponding standard deviations (or equivalently, variances) from the selected studies. However, not all studies report these quantities directly. Instead, studies may report mean and confidence interval, *p*-value, median, minimum and maximum, range or interquartile range (IQR). As another example, when Bayesian methods were employed in the data analysis, posterior means and 95 % credible intervals are usually reported.

If the mean and standard deviation are not directly reported in the publication, these need to be estimated from the other reported summary statistics. Wiebe et al. [1] describe several methods, including algebraic and approximate algebraic recalculations, to obtain standard deviation estimates from confidence levels, *t*-test or *F*-test statistics, and *p*-values. Based on descriptive statistics (such as the median, minimum and maximum, range, or the IQR), the *ad*-*hoc* approach is a study-level imputation. For instance, the sample median is often used as the estimate of the sample mean assuming symmetric distribution, and the sample standard deviation is commonly estimated by either $\frac{range}{4}$ or $\frac{IQR}{1.35}$.

Hozo et al. [2] proposed a simple alternative method for estimating the sample mean and the sample standard deviation from the median, minimum, maximum, and the size of the sample. Another alternative method was proposed by Bland [3] estimating these quantities based on the minimum, first quartile, median, third quartile, maximum, and sample size. Recently, Wan et al. [4] proposed a method that improved estimation of the sample standard deviation based on the median, minimum, maximum, and the size of the sample. Wan et al. [4] also provided a method for estimating the standard deviation based on the median, the quartiles, and the size of the sample.

In this paper, we propose an Approximate Bayesian Computation (ABC) approach for estimating the mean and standard deviation. This method produced more precise estimates of true study-specific mean and standard deviation as sample size increases and it also accommodates various distributions.

In ‘Methods’ section we summarize the methods of Hozo et al. [2], Bland [3] and Wan et al. [4] and describe our proposed ABC method. In ‘Results’, we describe and report the findings of the simulation studies comparing the performance of these methods. We used the statistical software R in performing all statistical programming related to the implementation of the various methods, analysis, and simulations.

## Methods

We denote the sample summary statistics as follows: minimum (*x*_min_), first quartile (*x*_Q1_), median (*x*_med_), third quartile (*x*_Q3_), maximum (*x*_max_), and sample size (*n*). We also consider the following three scenarios of available summary statistics. The first scenario (S1) assumes availability of only the minimum, median, maximum and sample size (S1 = {*x*_min_, *x*_med_, *x*_max_, *n*}). The second scenario (S2) assumes additionally having estimates of the first and third quartiles (S2 = {*x*_min_, *x*_Q1_, *x*_med_, *x*_Q3_, *x*_max_, *n*}). The third scenario (S3) assumes having the median, first quartile, third quartile, and sample size (S3 = {*x*_Q1_, *x*_med_, *x*_Q3_, *n*}).

### Method of Hozo et al.

The method by Hozo et al. [2] makes no assumption on the distribution of the underlying data. Hozo et al. proposed the following formulas for estimating the mean and variance under S1 = {*x*_min_, *x*_med_, *x*_max_, *n*}

1 $$ \overline{x}\approx \left\{\begin{array}{cc}\hfill \frac{x_{min}+2{x}_{med}+{x}_{max}}{4}\hfill & \hfill n\le 25\hfill \\ {}\hfill {x}_{med}\hfill & \hfill n>25\hfill \end{array}\right. $$

and

2 $$ {S}^2\approx \left\{\begin{array}{cc}\hfill \frac{1}{12}\left({\left(\frac{x_{min}+2{x}_{med}+{x}_{max}}{4}\right)}^2+\left({x}_{max}-{x}_{min}\right)\right),\hfill & \hfill n\le 15\hfill \\ {}\hfill {\left(\frac{x_{max}-{x}_{min}}{4}\right)}^2\hfill & \hfill 15<n\le 70\hfill \\ {}\hfill {\left(\frac{x_{max}-{x}_{min}}{6}\right)}^2\hfill & \hfill n>70\hfill \end{array}\right. $$

The Hozo et al. approach utilizes different formulas for estimating the mean and variance depending on the sample size *n*. When sample size is between 26 and 70, Hozo et al.’s formulas in Eqs. (1) and (2) are exactly the same as mean and variance formulas by the *ad*-*hoc* approach mentioned above.

### Method of Bland

Similar to Hozo et al., the method by Bland [3] also makes no assumption on the distribution of the underlying data. Bland [3] extended the method of Hozo et al. by adding first quartile (x_Q1_) and third quartile (x_Q3_) to S1. Bland’s method provides formulas to estimate the mean and variance under S2 = {*x*_min_, *x*_Q1_, *x*_med_, *x*_Q3_, *x*_max_, *n*}. While Hozo et al. used the sample size to decide the formula to be employed in estimating the mean and variance, the method by Bland incorporates the sample size in the proposed formulas:

3 $$ \begin{array}{c}\hfill \overline{x}=\frac{\left(n+3\right){x}_{min}+2\left(n-1\right)\left({x}_{Q1}+{x}_{med}+{x}_{Q3}\right)+\left(n+3\right){x}_{max}}{8n}\hfill \\ {}\hfill = \frac{\left({x}_{min}+2{x}_{Q1}+2{x}_{med}+2{x}_{Q3}+{x}_{max}\right)}{8} + \frac{3\left({x}_{min}+{x}_{max}\right)-2\left({x}_{Q1}+{x}_{med}+{x}_{Q3}\right)}{8n}\hfill \end{array} $$

4 $$ \approx \frac{\left({x}_{min}+2{x}_{Q1}+2{x}_{med}+2{x}_{Q3}+{x}_{max}\right)}{8} $$

and

5 $$ {S}^2=\frac{\left(n+3\right)\left({x}_{min}^2+2{x}_{Q1}^2+2{x}_{med}^2+2{x}_{Q3}^2+{x}_{max}^2\right)+8\left({x}_{min}^2+{x}_{max}^2\right)}{16n}\kern0.75em +\frac{\left(n-5\right)\left({x}_{Q1}\left({x}_{min}+{x}_{med}\right)+{x}_{Q3}\left({x}_{med}+{x}_{max}\right)\right)}{n} $$

6 $$ \approx \frac{\left({x}_{min}^2+2{x}_{Q1}^2+2{x}_{med}^2+2{x}_{Q3}^2+{x}_{max}^2\right)}{16}+\frac{x_{Q1}\left({x}_{min}+{x}_{med}\right)+{x}_{Q3}\left({x}_{med}+{x}_{max}\right)}{8}-{\overline{x}}^2. $$

Note that in Eq. (6), the third term is the squared value of mean estimate using Eq. (4). As pointed by Wan et al., the 2nd term in Eq. (3) can be ignored when sample size is large. Thus, after dropping the second term in (3), the estimators in (4) and (6) do not involve the sample size (*n*). Wan et al. proposed alternative estimators under S2, as described in next subsection.

### Method of Wan et al.

The method by Wan et al. [4] is based on order statistics and it assumes that the outcome is normally distributed. They proposed estimation formulas for the mean and standard deviation under the three scenarios, S1, S2, and S3, of available summary statistics, although their main focus was on improvement of standard deviation estimation.

For estimation of mean, Wan et al. proposed in S1 the same formula (1) by Hozo et al. [2], and in S2 the same formula (3) by Bland [3]. In S3 = {*x*_Q1_, *x*_med_, *x*_Q3_, *n*}, they proposed the following new estimation formula for mean:

7 $$ \overline{x}\approx \frac{\left({x}_{Q1}+{x}_{med}+{x}_{Q3}\right)}{3}. $$

For estimation of standard deviation, Wan et al. proposed the following formulas:

8 $$ \begin{array}{cc}\hfill S\approx \frac{\left({x}_{max}-{x}_{min}\right)}{2{\Phi}^{-1}\left(\frac{n-0.375}{n+0.25}\right)}\hfill & \hfill \mathrm{in}\;\mathrm{S}1,\hfill \end{array} $$

9 $$ \begin{array}{cc}\hfill S\approx \frac{1}{2}\left(\frac{\left({x}_{max}-{x}_{min}\right)}{2{\Phi}^{-1}\left(\frac{n-0.375}{n+0.25}\right)}\right)+\frac{1}{2}\left(\frac{\left({x}_{Q3}-{x}_{Q1}\right)}{2{\Phi}^{-1}\left(\frac{0.75n-0.125}{n+0.25}\right)}\right)\hfill & \hfill \mathrm{in}\;\mathrm{S}2,\hfill \end{array} $$

10 $$ \begin{array}{cc}\hfill S\approx \frac{\left({x}_{Q3}-{x}_{Q1}\right)}{2{\Phi}^{-1}\left(\frac{0.75n-0.125}{n+0.25}\right)}\hfill & \hfill \mathrm{in}\ \mathrm{S}3\hfill \end{array} $$

where Φ^− 1^ is the inverse of cumulative standard normal distribution.

Note that the standard deviation estimator in S2, Eq. (9), is simply the weighted average of those in S1 and S3, per Eqs. (8) and (10), respectively. The Wan et al. estimator of the standard deviation is based on normality assumption and uses approximation of expected values of the order statistics.

### Simulation-based method via Approximate Bayesian Computation (ABC)

We propose a simulation-based method using the Approximate Bayesian Computation (ABC) technique to estimate the sample mean and standard deviation.

Bayesian inference needs likelihood functions as well as priors for the parameters in the model. Given a likelihood function, f(θ|D), where θ denotes parameter of interest and D denotes observed data, and prior distribution, p(θ), on the parameter space, Θ, our statistical inference is based on posterior distribution of θ, p(θ|D)∝f(θ|D)p(θ). In some situations, the likelihood function is analytically or computationally intractable. In meta-analysis, we combine selected studies with respect to a certain clinical outcome. However, the datasets of these studies are usually not accessible. Although we can construct a likelihood function based on the probability model, we cannot evaluate the likelihood function due to unavailability of all data points. Using the Approximate Bayesian Computation (ABC) approach, the likelihood can be replaced by a comparison of summary statistics from the observed data and those from simulated data using a distance measure. The ABC methodology was introduced by Tavaré et al. [5] in population genetics using a simple rejection algorithm in order to avoid the computation of the likelihood function via a simulation from a specific distribution. Marin et al. [6] provided an extensive review of several ABC methods.

Table 1 describes how to use ABC method for estimation of the mean and standard deviation using summary statistics. The first step is to generate a set of candidate values for parameters, θ*, from a specific prior distribution, p(θ). The second step is to generate pseudo data, D*, from the likelihood function f(θ*). The third step is to decide whether θ* is accepted or not. This decision depends on the distance between summary statistics of the observed data, S(D), and those of simulated data, S(D*) denoted by ρ(S(D),S(D*)), where ρ(•,•) is a distance measure. In our application of ABC, we used the Euclidean distance measure. If ρ(S(D),S(D*)) is smaller than a fixed tolerance value ε (i.e., ρ(S(D),S(D*)) < ε), then θ* is accepted, otherwise it is rejected. Steps 1–3 are repeated a large number of times (e.g., *N* = 20,000) in order to obtain multiple sets of θ* for the inference. Instead of setting a small tolerance value ε, we can alternatively select a fixed number of sets of θ* corresponding to an acceptance percentage. For example, with acceptance percentage of 0.1 % and *N* = 50,000, we select 50 values of θ* corresponding to the top 0.1 % with smallest Euclidean distance. The fundamental idea of ABC is that a good approximation of the posterior distribution can be obtained using summary statistics, S(D), and a fixed small tolerance value ε (or a pre-specified acceptance percentage).

**Table 1:** Scheme of ABC and required settings for simulation-based estimation

ABC steps		
1	θ* ~ p(θ); generate θ* from prior distribution	
2	D* ~ f(θ*); generate pseudo data	
3	Compute summary statistics, S(D*), from D* and compare with given summary statistics, S(D).	
	If ρ(S(D*),S(D)) < ε, then θ* is accepted	
Repeat steps 1–3 many times to obtain enough number of accepted θ* for statistical inference		
Settings for simulation-based estimation of mean and standard deviation		
	Specify	Example
A	Underlying data distribution. (e.g.: normal, log-normal, exponential)	Normal (μ, σ)
		Given the nature of the outcome variable, an educated decision about the underlying distribution can be made.
B	Prior uniform distribution for each underlying parameter.	For μ, use U(X_min_, X_max_) in S1, or
		U(X_Q1_, X_Q3_) in S2 and S3.
		For σ, use U(0, L) where L denotes some large number beyond X_max_in S1 or X_Q3_ in S2 and S3.
C	Acceptance percentage and number of iterations	Acceptance of 0.1 % and 50,000 or 100, 000 iterations.

In order to apply ABC algorithm to estimate mean and standard deviation using reported summary statistics, the first step is to choose a distribution to be used for generating data. (Table 1, lower panel.) Given a set of summary statistics and the nature of outcome variable, an educated decision about the distribution can be made. For example, if clinical outcome is some score of health-related quality of life (e.g. The Expanded Prostate Cancer Index Composite (EPIC) score ranging from 0 to 100), then such a variable is bounded and in this case we can use beta distribution. For unbounded variable we can choose either normal or log-normal distribution. When variable is change between two measurements, normal distribution is a good choice. When variable is either percentage or strictly positive, then log-normal, exponential, or Weibull are good choices. Next step is to determine prior distributions to generate parameters of a chosen distribution. For example, if normal or log-normal distribution is chosen we need to specify prior distributions for μ and σ. For Weibull distribution, prior distributions for shape and scale parameters are needed. For beta distribution, prior distributions for two shape parameters are needed. Usual choice for prior is uniform distribution with relative wide range. When a chosen distribution belongs to location-scale family, we can use an educated guess for location parameter μ. Instead of uniform distribution with huge range, we can use given summary statistics such as minimum (x_min_) and first quartile (x_Q1_) (maximum (x_max_) and third quartile (x_Q3_) for lower bound (upper bound) of uniform distribution. Prior distributions for shape and scale parameters are uniform between zero and some large number.

The estimates of mean and standard deviation by ABC are obtained based on accepted parameter values. For instance, when we consider normal distribution, average of accepted values for μ is the estimated mean; likewise, the average of accepted values for σ is the estimated standard deviation. For non-normal distributions, estimates of the mean and standard deviation can be obtained from a ‘plug-in method’ or ‘simulation’. Both approaches give comparable estimates. The plug-in method consists of replacing means of accepted parameter values into the corresponding formulas for the mean and standard deviation. For example, the beta distribution has mean α/(α + β) and variance αβ/[(α + β)^2^(α + β + 1)]. We obtain estimates of the mean and standard deviation by replacing in these formulas α and β with mean of accepted values for these parameters.

The simulation approach consists of obtaining the mean and standard deviation from simulated samples using each set of accepted parameter values. For example, in beta distribution, given a set of accepted values of α and β, we generate pseudo data of the same sample size and calculate the mean and standard deviation from pseudo data. We repeat this procedure for all sets of accepted parameter values. The simulation estimates of the mean and standard deviation are the average of means and average of standard deviations, respectively.

## Results

### Designs of simulation studies

In order to facilitate comparison between our ABC method and existing methods, the parameters of our simulation studies were set to be similar to that by Hozo et al. and Wan et al. for the three different scenarios of available descriptive statistics.

Under S1, we compare ABC to Hozo et al. and Wan et al. Under S2, we compare ABC, Bland and Wan et al. methods. And under S3, we compare ABC and Wan et al. methods. In addition, we examine the effect of skewness in estimation performance using log-normal and beta distributions.

Under S1, we use the same five distributions which both Hozo et al. and Wan et al. simulated: normal distribution with mean 50 and standard deviation 17, N(50,17); log-normal distribution with location parameter = 4 and scale parameter = 0.3, LN(4,0.3); Weibull distribution with shape parameter = 2 and scale parameter = 35, Weibull(2,35), beta distribution with two shape parameters 9 and 4, Beta(9,4); and exponential distribution with mean = 10, Exp(10).

Under S2, we use log-normal distribution with same location parameter value of 5 and three different scale parameter values (0.25, 0.5, and 1) in order to evaluate effect of skewness. We also use three beta distributions, Beta(5,2), Beta(1,3), and Beta(0.5,0.5), to examine effect of skewness and bimodality in estimation for bounded data distribution.

Under S3, we use four distributions in S1 (lognormal, beta, exponential and Weibull) to investigate further the effect the choice of descriptive statistics for the standard deviation estimation.

In each scenario we consider 10 sample sizes (*n* = 10, 40, 80, 100, 150, 200, 300, 400, 500, 600). We obtain a sample of *n* observations from a particular distribution, and compute the sample mean (true $\overline{x}\Big)$ and sample standard deviation true S). Using the different methods (Hozo et al. Bland, Wan et al. and ABC) we obtain the various estimates of the mean and standard deviation from the corresponding sample descriptive statistics. The relative errors (REs) are calculated as follows:

11 $$ RE\  of\  mean=\frac{\left( estimated\ \overline{x} - true\kern0.5em \overline{x}\right)}{true\ \overline{x}}, $$

and

12 $$ RE\  of\  standard\  deviation=\frac{\left( estimated\ S - true\ S\right)}{true\kern0.5em S}. $$

For each sample size *n*, we repeat this procedure 200 times to obtain average relative errors (AREs).

In the simulations, we set acceptance percentage 0.1 % and 20,000 total number of iterations for ABC method. Hence, we obtain 20 accepted parameter values for a specific distribution. Prior distributions for each distribution in the ABC model for the simulation are described in Table 2.

**Table 2:** Prior distributions for ABC in the simulation studies

Distribution	Parameter 1	Prior for parameter 1	Parameter 2	Prior for parameter 2
Normal (S1)	μ	Uniform (X_min_, X_max_)	σ	Uniform(0,50)
Normal (S2)	μ	Uniform (X_Q1_, X_Q3_)	σ	Uniform(0,50)
Normal (S3)	μ	Uniform (X_Q1_, X_Q3_)	σ	Uniform(0,50)
Log-normal (S1)	μ	Uniform (log(X_min_), log(X_max_))	σ	Uniform(0,10)
Log-normal (S2)	μ	Uniform (log(X_Q1_), log(X_Q3_))	σ	Uniform(0,10)
Log-normal (S3)	μ	Uniform (log(X_Q1_), log(X_Q3_))	σ	Uniform(0,10)
Exponential	λ	Uniform(0,40)	-	-
Beta	α	Uniform(0,40)	β	Uniform(0,40)
Weibull	λ	Uniform(0,50)	κ	Uniform(0,50)

### Results of simulation studies

In the simulation studies we compare estimation performance of the various methods in terms of average relative error (ARE) for estimating mean and standard deviation. In the next three subsections we present comparison of methods for standard deviation estimation. In the last subsection, we present comparison among methods for mean estimation.

### Comparison of Hozo et al., Wan et al., and ABC in S1 for standard deviation estimation

**Fig. 1:**
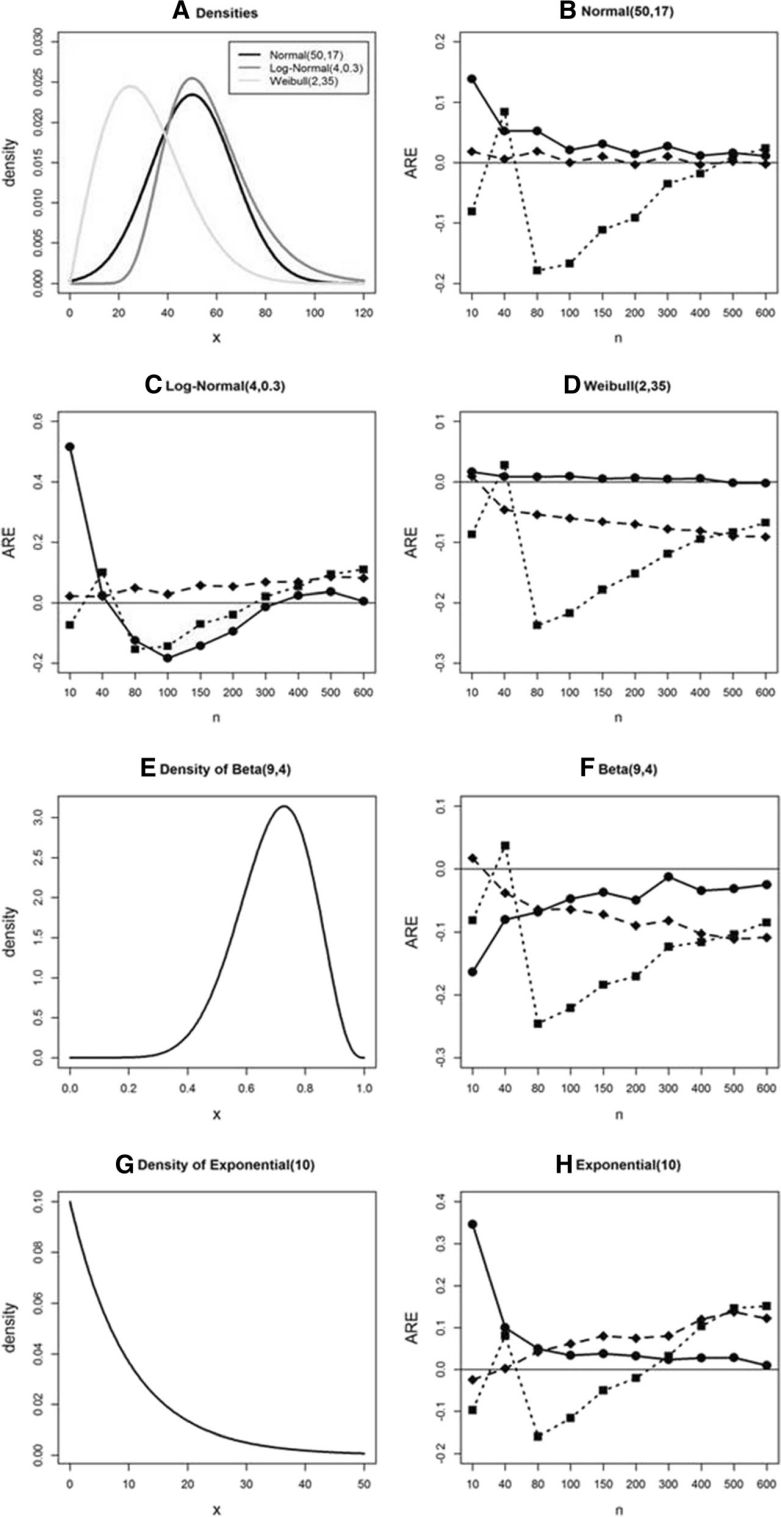
Average relative error (ARE) comparison in estimating sample standard deviation under S1 using simulated data from five parametric distributions. **a**, **e**, **g**: Density plots for normal, log-normal, Weibull, beta, and exponential distributions. **b**, **c**, **d**, **f**, **h**: AREs for 3 methods using simulated data from normal, log-normal, Weibull, beta, and exponential distributions. Hozo et al. (*solid square* with *dotted line*), Wan et al. (*solid diamond* with *dashed line*), and ABC (*solid circle* with *solid line*) methods

In Fig. 1 we show AREs in estimating standard deviation for the three methods as a function of sample size under simulated data from the selected five distributions. The corresponding densities are displayed in Fig. 1a (normal, log-normal, and Weibull), 1e (beta) and 1g (exponential). Under the normal distribution (Fig. 1b) in S1 (that is, when *x*_min_, *x*_med_, *x*_max_, *n* are available), while the Hozo et al. method (solid square linked with dotted line) shows large average relative errors for sample size less than 300, the Wan et al. method (solid diamond linked with dashed line) shows quite good performance over all sample sizes. The ABC method (solid circle linked with solid line) shows decreasing error as sample size increases, with AREs close to that for the Wan et al. method for *n* ≥80.

Under the log-normal distribution (Fig. 1c), the Hozo et al. method shows better performance between sample sizes of 200 and 400. The Wan et al. method still shows good performance, though there is a tendency of AREs moving away from zero as sample size increases. The ABC method has slightly worse performance than does the Wan et al. method when sample size is less than 300. It is the best when sample size is greater than 300, and it is the worst for small sample size around *n* = 10.

For Weibull data (Fig. 1d), the ABC method is the best, showing very small AREs close to zero over all sample sizes. The Wan et al. method clearly shows that ARE moves away from zero as sample size increases.

For data from beta or exponential distributions (Fig. 1f and h), the ABC method performed best, showing AREs approaching zero as sample size increases. The Wan et al. method shows an opposite tendency of increasing ARE as sample size increases.

### Comparison of Bland, Wan et al., and ABC in S2 for standard deviation estimation

**Fig. 2:**
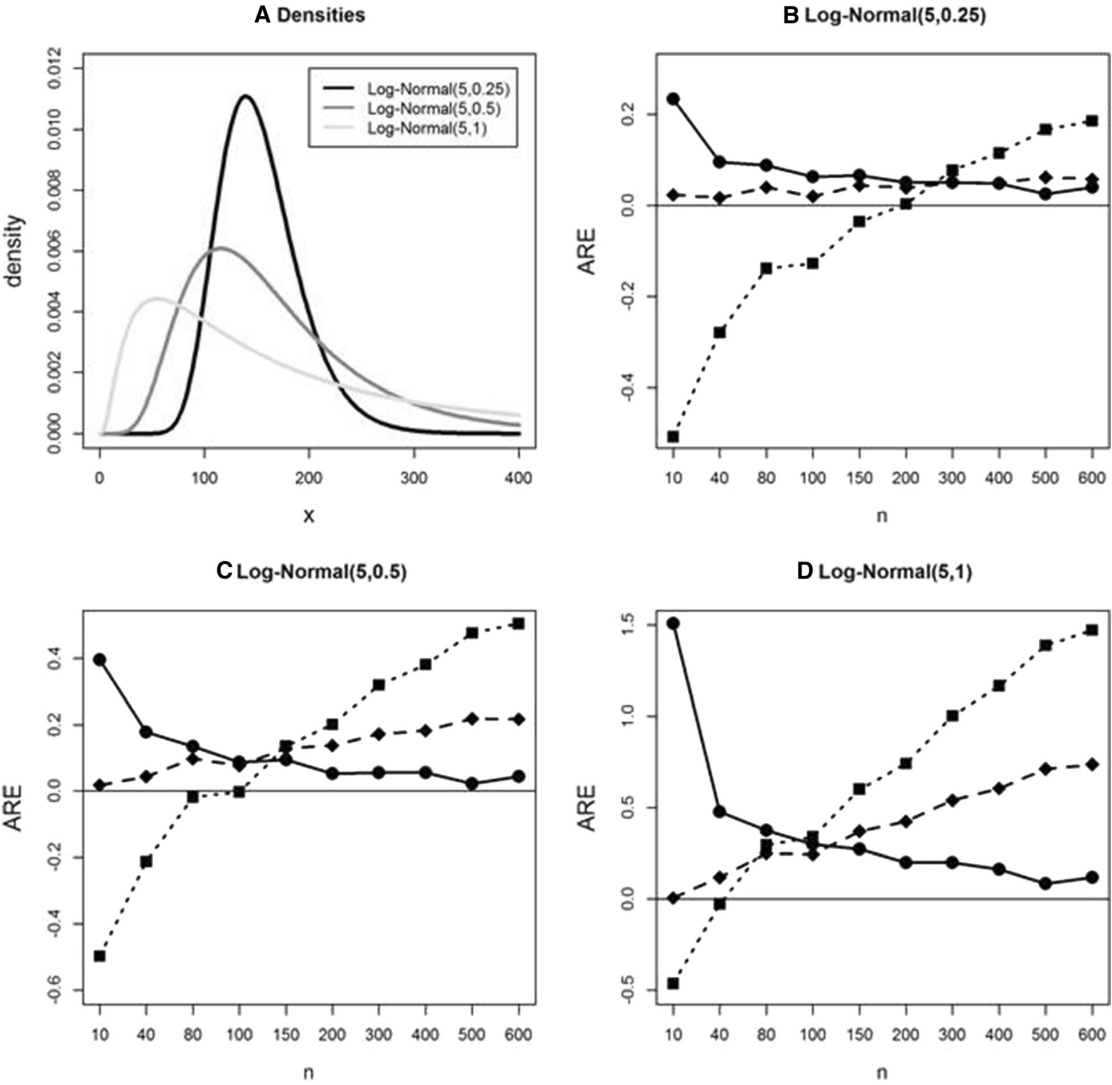
Average relative error (ARE) comparison in estimating sample standard deviation under S2 using simulated data from log-normal distributions. **a**: Density plots for 3 log-normal distributions. **b**, **c**, **d**: AREs for 3 methods using simulated data from the same 3 log-normal distributions. Bland (*solid square* with *dotted line*), Wan et al. (*solid diamond* with *dashed line*), and ABC (*solid circle* with *solid line*) methods

In this simulation we compare estimation of standard deviation under these methods in S2 (that is, when *x*_min_, *x*_Q1_, *x*_med_, *x*_Q3_, *x*_max_, and *n* are available) and examine the effect of violation of normality using the log-normal distribution. We consider three log-normal distributions with the same location parameter value but three different scale parameters (Fig. 2a). For LN(5,0.25), the Wan et al. and ABC methods have a similar small ARE. Bland’s method shows argely underestimates for small sample size, and the ARE keeps increasing as sample size increase. Note that AREs increase when sample size is over 200. As data are simulated from more skew to the right distributions (Fig. 2c and d), we see large estimation errors in Bland and Wan et al. methods. Wan et al. method shows increasing ARE as sample size increases. Using the Bland method the true study-specific standard deviation is underestimated (large negative ARE) in small sample size *n* and overestimated (large positive ARE) in large *n*. The AREs of the ABC method are large with small sample size when skewness increases; however, AREs of the ABC method become smaller and approaches zero as sample size increases.

**Fig. 3:**
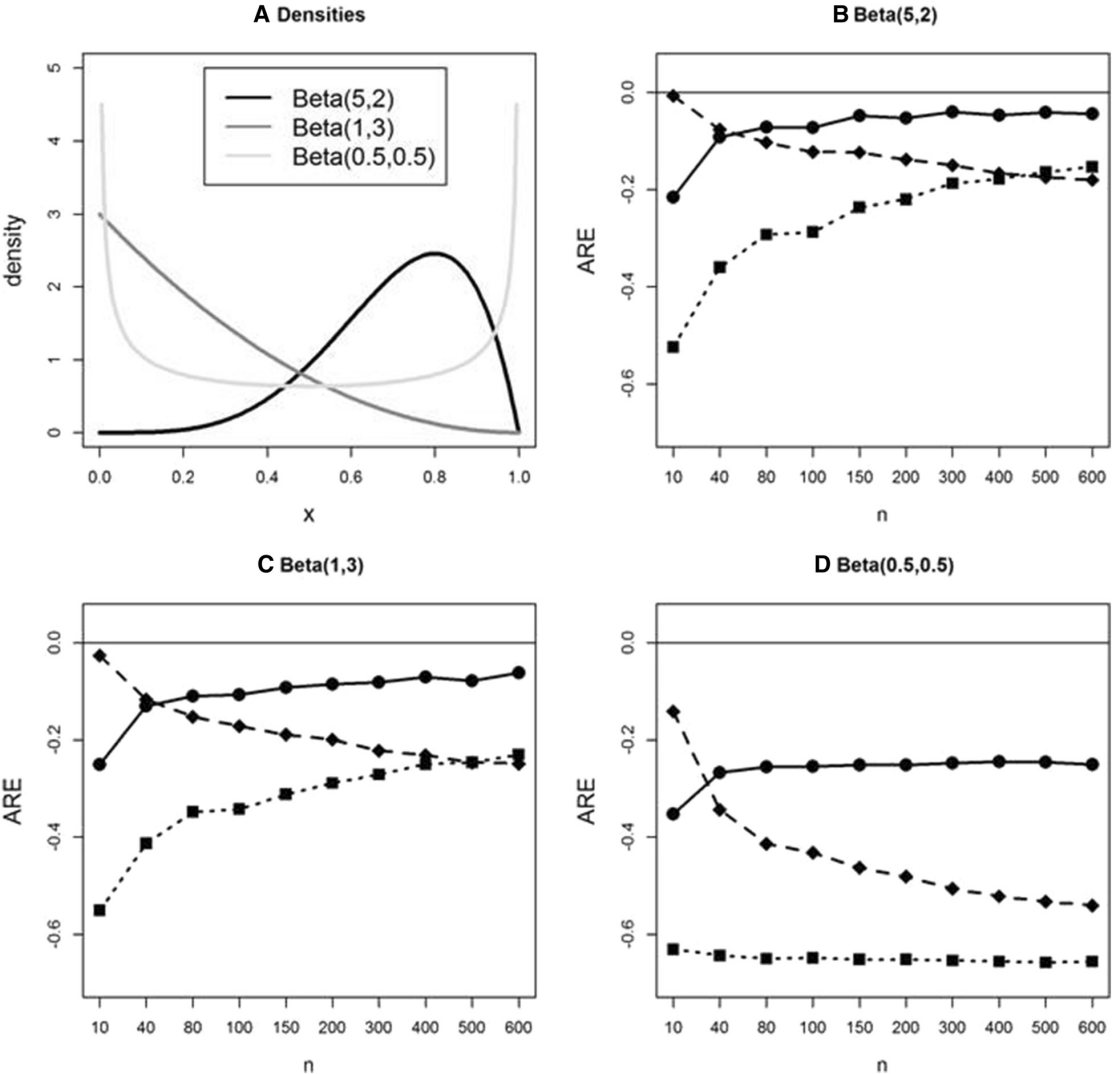
Average relative error (ARE) comparison in estimating sample standard deviation under S2 using simulated data from beta distributions. **a**: Density plots for 3 beta distributions. **b**, **c**, **d**: AREs for 3 methods using simulated data from the same 3 beta distributions. Bland (*solid square* with *dotted line*), Wan et al. (*solid diamond* with *dashed line*), and ABC (*solid circle* with *solid line*) methods

We also examine the performance of these methods when data are simulated from three beta distributions. (Fig. 3) In this simulation study, we investigate the effect of bimodality as well as skewness for bounded data. For all methods underestimation of study-specific true standard deviation is depicted, with ABC performing best for *n* >40. Under skewed distributions (Fig. 3b and c) the Bland and ABC methods show the same pattern, however ABC shows much better performance since ARE approaches zero with increasing sample size. When the underlying distribution is bimodal (Fig. 3d), all three methods show large underestimation, although ABC continues performing best for *n* >40, showing smaller AREs.

### Comparison of Wan et al. and ABC in estimating standard deviation under S1, S2, and S3

**Fig. 4:**
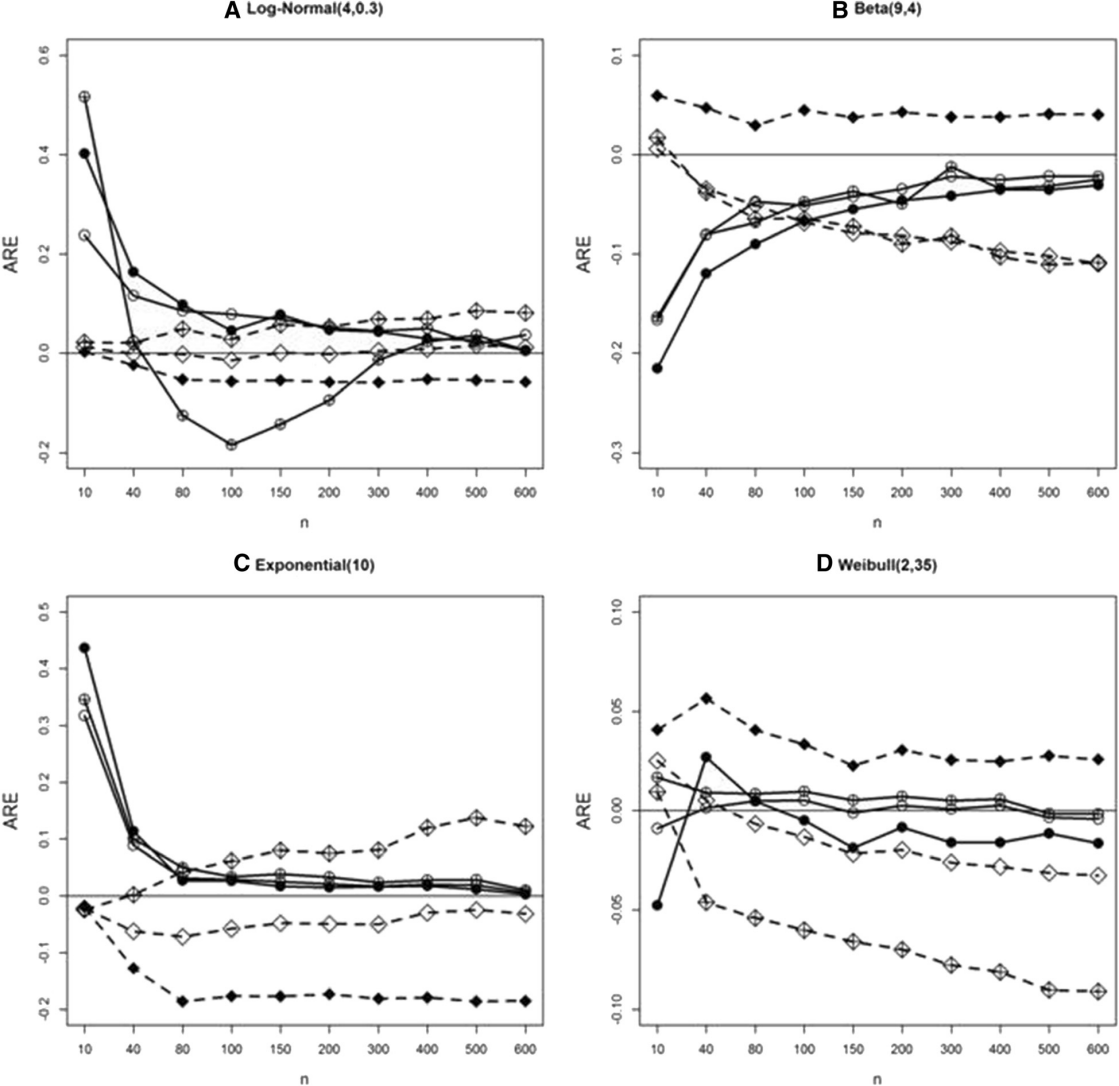
Average relative error (ARE) comparison in estimating sample standard deviation under S1, S2 and S3 using simulated data from four parametric distributions. **a**, **b**, **c**, **d**: AREs for 3 methods using simulated data from log-normal, beta, exponential, and Weibull distributions. Wan et al. (*dashed line* and *crossed diamond* for S1, *diamond* for S2, and *solid diamond* for S3); and ABC (*solid line* and *crossed circle* for S1, *circle* for S2, and *solid circle* for S3) methods

Here we simulate data in S1, S2, and S3 under four distributions: log-normal, beta, exponential, and Weibull. In Fig. 4, crossed symbols denote S1, open symbols S2, and solid symbols S3. Circle and diamond denotes the ABC method and the Wan et al., respectively. Under the several distributions, AREs for the ABC method converge toward zero as sample size increases for the three scenarios, while Wan et al. fail to show this pattern.

### Comparison of methods for mean estimation

**Fig. 5:**
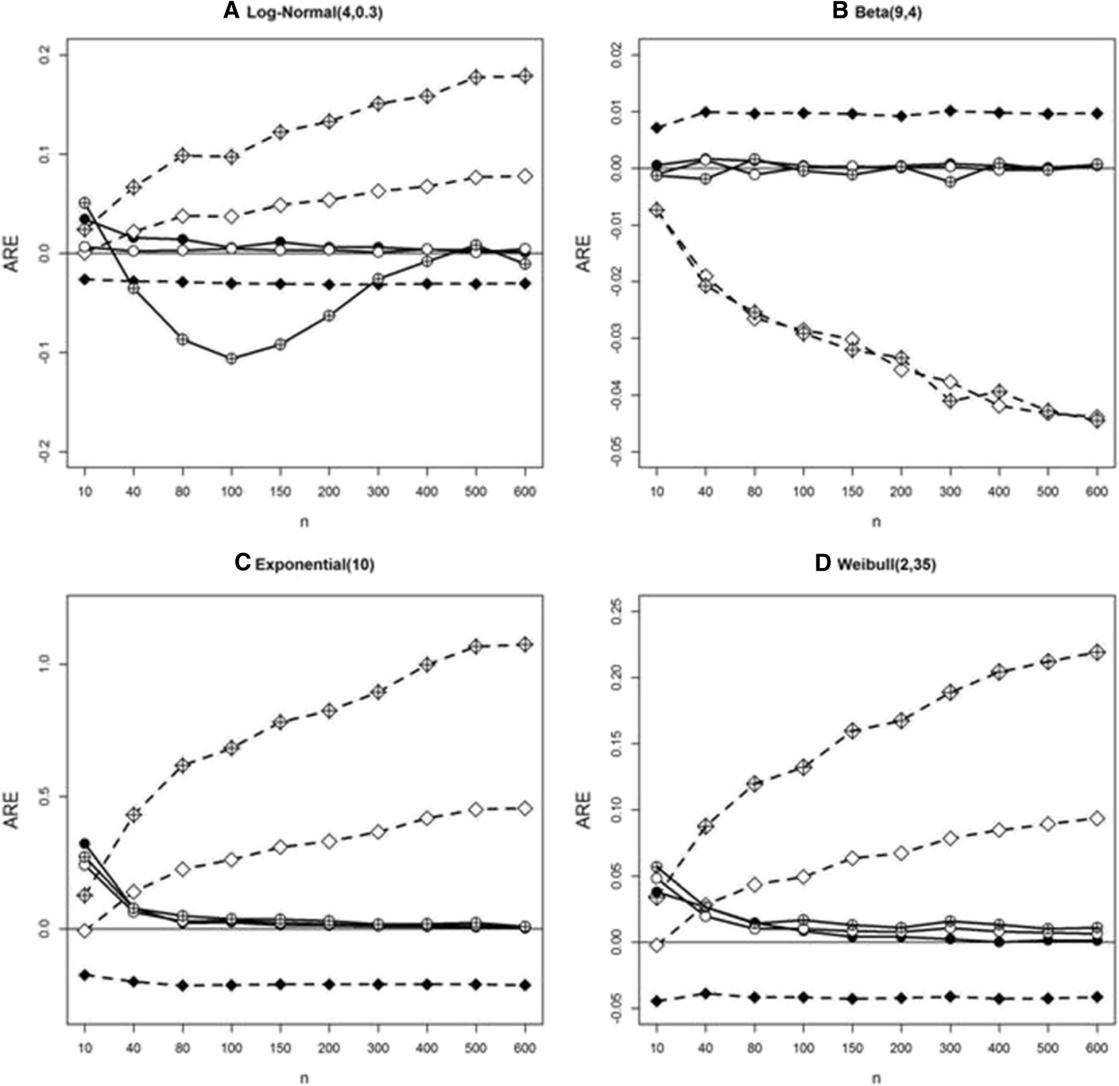
Average relative error (ARE) comparison in estimating sample mean under S1, S2 and S3 using simulated data from four parametric distributions. **a**, **b**, **c**, **d**: AREs for 3 methods using simulated data from log-normal, beta, exponential, and Weibull distributions. Wan et al. (*dashed line* and *crossed diamond* for S1, *diamond* for S2, and *solid diamond* for S3); and ABC (*solid line* and *crossed circle* for S1, *circle* for S2, and *solid circle* for S3) methods

We compare AREs for mean estimation between the Wan et al. and ABC methods. Note that the mean formula is the same between Wan et al. [4] and Hozo et al. [2] under S1, and between Wan et al. and Bland [3] under S2. Figure 5 indicates that our ABC method is superior in estimating the mean when sample size is greater than 40 for all scenarios. Under the log-normal in S1 the pattern of AREs of mean estimates for ABC in S1 is similar to that of standard deviation estimate for ABC (see Fig. 1c). However, as sample size increases the ARE approaches zero.

## Discussion

The main factor that has a huge influence in the performance of the three methods is the assumed parametric distribution, especially when the samples are drawn from a skewed heavy-tailed distribution. Since inputs for the estimation of the standard deviation in S1 are minimum value (x_min_), median (x_med_), and maximum value (x_max_), the two extreme values vary a lot from data set to data set. The inferior performance of the ABC method under normal, log-normal, and exponential distributions with small sample size can be explained by erratic behavior of two extreme values as an input. However, as sample size increases, the ARE of ABC method becomes small and ABC is better than the other methods. The Wan et al. method is based on a normal distribution assumption. Thus, it performs well under the normal distribution or any distribution close to symmetric in shape (e.g., beta(4,4) is symmetric at 0.5). When the underlying distribution is skewed or heavy-tailed, although Wan et al. method incorporates sample size into the estimation formulas, the AREs keep deviating from zero as sample size increases.

In order to perform ABC we need to choose an underlying distribution model. This choice can be based on an educated guess. For instance, when outcome is related to distribution with positive support, there are several distributions to be considered, such as log-normal, Weibull, or exponential. In this situation we rely on model selection (i.e. distribution selection in our context) while we apply the ABC method. Bayesian model selection is usually based on either the Bayes factor or marginal posterior probability of model. Let M_1_ and M_2_ be two models according to two different distributions (e.g., normal and beta distributions). The Bayes factor is defined as

13 $$ {B}_{12}=\frac{P\left({M}_1\Big|D\right)/P\left({M}_2\Big|D\right)}{P\left({M}_1\right)/P\left({M}_2\right)}, $$

where P(M_i_) is the prior and P(M_i_|D) is the marginal posterior distribution of model M_i_, *i* = 1,2, and D denotes data. When we assume that P(M_1_) = P(M_2_) = 0.5 then the Bayes factor is a ratio of two marginal posterior distributions of the model, P(M_1_|D)/P(M_2_|D). In the ABC approach, data are not available so we replace summary statistics, S, for D. The Bayes factor and marginal posterior probability of the model can be approximated by the acceptance frequency for each model (i.e., distribution) in the ABC. It can be extended when we consider more than two distributions for comparison. When we have K distributions (K >2) to be considered as candidate distribution, we perform model selection within ABC and calculate corresponding marginal posterior model probabilities (P(M_k_|S), k = 1,…K). Then we choose the distribution with the highest marginal posterior model probability among K candidate distributions. We performed a small simulation to see whether this approach is reliable for selecting appropriate distributions for ABC. We generated samples of size 400 from beta(9,4). We computed marginal posterior model probabilities for beta, P(M_1_|S), and for normal, P(M_2_|S). Note that P(M_2_|S) = 1-P(M_1_|S), when only two distributions are considered. We repeated 200 times to tabulate how many times beta distribution is chosen, as well as to get the estimates of the average of marginal posterior model probabilities. The beta distribution was chosen 157 times among 200 repeats (78.5 %), average of P(M_1_|S) was 0.63 and average P(M_2_|S) was 0.37. The AREs of estimated standard deviation using beta and normal distributions were −0.0216 and 0.0415, respectively. The ARE of estimated mean using the beta distribution was 0.00068 and it was quite smaller than that of the normal distribution (0.0118). These results indicate that the distribution selection procedure works well. In real application, we would test candidate distributions using the summary data available, and select the distribution with largest posterior model probability, P(M|S). For example, we generated a sample of size *n* = 400 from beta(9,4). Summary sample statistics were 0.6184 (Q_1_), 0.6989 (median), 0.7904 (Q_3_), 0.6961(mean), and 0.1231(standard deviation). Assuming available Q_1_, median, Q_3,_ and *n*, and desire to test between beta and normal distributions as the underlying distribution, we ran ABC for model selection. P(M|S) for beta distribution was 65 and 35 % for normal. Thus, we would select the beta distribution.

In our simulation for the ABC method, we set an acceptance percentage of 0.1 % and *N* = 20,000 iterations, given the large number of settings. In real application we suggest using *N* = 50,000 or more iterations and acceptance percentage 0.1 % to get enough accepted parameter values for reliably estimating the mean and standard deviation. We conducted sensitivity analysis for examining impact of value of acceptance percentage and the number of iterations on AREs (Additional file 1). We used normal distribution with mean 50 and standard deviation 17 in S1, S2, and S3. We considered three numbers of iterations (20,000, 50,000, and 100,000) and two acceptance percentages (0.1 and 0.01 %). All combinations of these settings show comparable performance in estimating standard deviation and mean with ARE approaching zero as sample size increases. In the standard deviation estimation, all combinations show comparable performance except in S2. In S2, 0.01 % acceptance percentage has lower AREs compared to those of 0.1 % acceptance percentage.

In order to examine impact of prior distribution setting on AREs we also conducted sensitivity analysis (Additional file 2). We used normal distribution with mean 50 and standard deviation 17 under S3, and considered three prior distributions, U(0,20), U(0,50), and U(0,100), for σ. Similar to the previous sensitivity analysis we used three numbers of iterations, 20,000, 50,000, and 100,000. We also reported AREs in estimating mean using these settings. In estimating SD, prior U(0,20) for σ gives negative AREs when sample size is <200 while other prior distributions (U(0,50) and U(0,100)) give positive AREs, regardless of the number of iterations. The opposite direction of AREs between U(0,20) and other prior distributions is related to distance between σ and upper bound of uniform distribution. Since true σ = 17 is close to upper bound 20, most accepted values for estimated SDs are lower than 17 and AREs are negative. For U(0,50) and U(0,100), majority of accepted values for estimated SDs are larger than 17 and AREs are positive. However, as sample size increases, AREs of all three prior distributions converge to zero. Note that estimation of means is not affected by prior distribution for σ.

In this paper we implement the ABC method using a simple rejection algorithm. We provide an example R code to help readers implement our simulation-based estimation method (Additional file 3). Other algorithms available include Markov chain Monte Carlo (ABC-MCMC; Marjoram et al. [7]) and sequential Monte Carlo (ABC-SMC; Toni et al. [8]). In future research, we plan to explore these methods for improving estimation of the mean and standard deviation. We also plan to conduct more thorough simulation study for evaluating performance of our simulation-based estimation method in complicated model selection and model averaging situation.

## Conclusion

We propose a more flexible approach than existing methods to estimate the mean and standard deviation for meta-analysis when only descriptive statistics are available. Our ABC method shows comparable performance to other methods as sample size increases in symmetric shape of the underlying distribution. However, our method performs much better than other methods when underlying distribution becomes skewed and/or heavy-tailed. The ARE of our method moves towards zero as sample size increases. Some studies applied Bayesian inference to conduct statistical analysis and reported a posterior mean and corresponding 95 % credible interval. In particular, a posterior mean typically does not locate at the center of the 95 % credible interval. In other situations, the maximum *a posteriori* probability (MAP) estimate is reported instead of a posterior mean. While other existing methods cannot be used for this situation, our ABC method is easily able to obtain estimates of the mean and standard deviation from these Bayesian summaries. In addition if we only have range or interquartile range and not the corresponding *x*_min_, *x*_med_, *x*_Q1_, *x*_Q3_, we can use ABC easily to get estimates for means and standard deviations.

## Additional files

Additional file 1:
**Sensitivity analysis for the number of iterations and acceptance percentage using Normal distribution with mean = 50 and SD = 17.** The plots in the top row display AREs for standard deviation estimate and mean estimate under S1. The plots in the middle row display AREs for standard deviation estimate and mean estimate under S2. The plots in the bottom row display AREs for standard deviation estimate and mean estimate under S3. In each plot six lines and symbols are displayed for the combination of the number of iterations and acceptance percentage. (JPEG 531 kb) Additional file 2:
**Sensitivity analysis for the number of iterations and prior distribution for σ using Normal distribution with mean = 50 and SD = 17.** The plots in the top row display AREs for standard deviation estimate and mean estimate under S3 with three different prior distributions for σ and 20,000 iterations. The plots in the middle row display AREs for standard deviation estimate and mean estimate under S3 with three different prior distributions for σ and 50,000 iterations. The plots in the bottom row display AREs for standard deviation estimate and mean estimate under S3 with three different prior distributions for σ and 100,000 iterations. (JPEG 377 kb) Additional file 3:
**R code example of ABC-based estimation.** This R code example generates a sample of *n* = 400 from a normal distribution with mean 50 and standard deviation 17, and uses sample estimates of X_min_, X_med_, X_max_ (that is, scenario S1) to illustrate how our simulation-based estimation method can be employed to obtain estimated sample mean and standard deviation. (DOCX 14 kb)

## References

[1] Wiebe N, Vandermeer B, Platt RW, Klassen TP, Moher D, Barrowman NJ (2006). A systematic review identifies a lack of standardization in methods for handling missing variance data. J Clin Epidemiol.

[2] Hozo SP, Djulbegovic B, Hozo I (2005). Estimating the mean and variance from the median, range, and the size of a sample. BMC Med Res Methodol.

[3] Bland M (2015). Estimating the mean and variance from the sample size, three quartiles, minimum, and maximum. Int J of Stat in Med Res.

[4] Wan X, Wang W, Liu J, Tong T (2014). Estimating the sample mean and standard deviation from the sample size, median, range and/or interquartile range. BMC Med Res Methodol.

[5] Tavaré S, Balding D, Griffith R, Donnelly P (1997). Inferring coalescence times from DNA sequence data. Genetics.

[6] Marin JM, Pudlo P, Robert CP, Ryder RJ (2012). Approximate Bayesian computational methods. Stat Comput.

[7] Marjoram P, Molitor J, Plagnol V, Tavaré S (2003). Markov chain Monte Carlo without likelihoods. Proc Natl Acad Sci U S A.

[8] Toni T, Ozaki YI, Kirk P, Kuroda S, Stumpf MPH (2012). Elucidating the in vivo phosphorylation dynamics of the ERK MAP kinase using quantitative proteomics data and Bayesian model selection. Mol Biosyst.

