# Supplementary figures and images for: Simulation-based estimation of mean and standard deviation for meta-analysis via Approximate Bayesian Computation (ABC)
Source: BMC Med Res Methodol. 2015 Aug 12;15:61. doi: 10.1186/s12874-015-0055-5 (PMC4542106; doi:10.1186/s12874-015-0055-5)

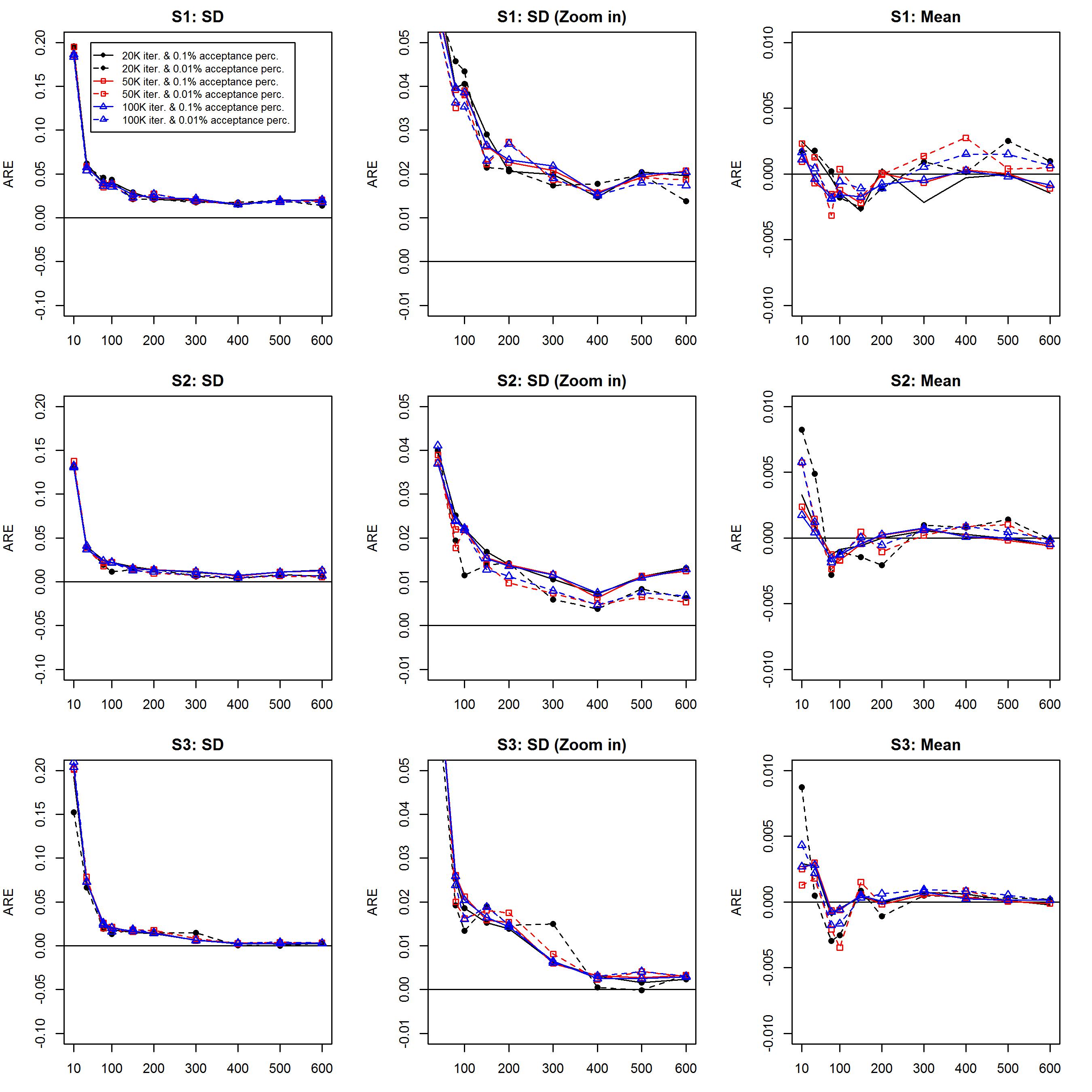

Supplement: Additional file 1: — Sensitivity analysis for the number of iterations and acceptance percentage using Normal distribution with mean = 50 and SD = 17. The plots in the top row display AREs for standard deviation estimate and mean estimate under S1. The plots in the middle row display AREs for standard deviation estimate and mean estimate under S2. The plots in the bottom row display AREs for standard deviation estimate and mean estimate under S3. In each plot six lines and symbols are displayed for the combination of the number of iterations and acceptance percentage. (JPEG 531 kb) [file 12874_2015_55_MOESM1_ESM.jpeg]

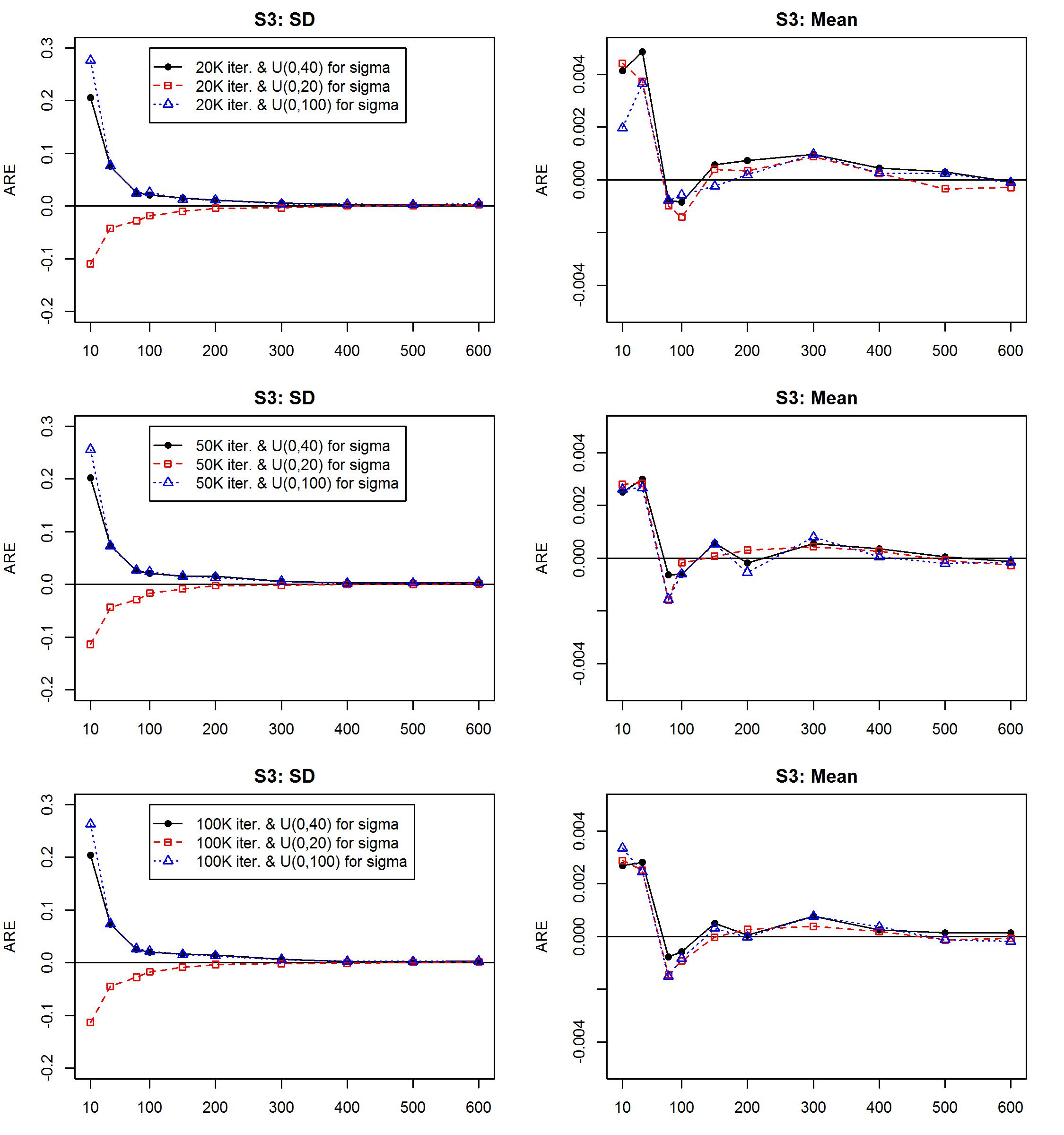

Supplement: Additional file 2: — Sensitivity analysis for the number of iterations and prior distribution for σ using Normal distribution with mean = 50 and SD = 17. The plots in the top row display AREs for standard deviation estimate and mean estimate under S3 with three different prior distributions for σ and 20,000 iterations. The plots in the middle row display AREs for standard deviation estimate and mean estimate under S3 with three different prior distributions for σ and 50,000 iterations. The plots in the bottom row display AREs for standard deviation estimate and mean estimate under S3 with three different prior distributions for σ and 100,000 iterations. (JPEG 377 kb) [file 12874_2015_55_MOESM2_ESM.jpeg]
