# Supplementary material for: Simulation-based estimation of mean and standard deviation for meta-analysis via Approximate Bayesian Computation (ABC)
Source: BMC Med Res Methodol. 2015 Aug 12;15:61. doi: 10.1186/s12874-015-0055-5 (PMC4542106; doi:10.1186/s12874-015-0055-5)
Supplement: Additional file 3: — R code example of ABC-based estimation. This R code example generates a sample of n = 400 from a normal distribution with mean 50 and standard deviation 17, and uses sample estimates of Xmin, Xmed, Xmax (that is, scenario S1) to illustrate how our simulation-based estimation method can be employed to obtain estimated sample mean and standard deviation. (DOCX 14 kb) [file 12874_2015_55_MOESM3_ESM.docx]

**Additional file 3.**

# Example assuming Normal distribution and S1 scenario (X_min_, X_med_, X_max_, and n are available)

# Generate a sample of n=400 from Normal(50, 17)

sample.data= round(rnorm(n,mu,sig), digits=2)

round(mean(sample.data),2);

round(sd(sample.data),2);

## Sample true Mean= 50.29 & SD= 16.47 (to be estimated by ABC-based method)

# Assume available summary statistics in S1

xmin=min(sample.data); xmin;

xmed=median(sample.data); xmed;

xmax=max(sample.data); xmax;

## Sample estimates: xmin=8.08, xmed=49.43 and xmax=105.1;

# Set of parameters for the ABC simulation-based estimates of Mean and SD ##

nb_simul.val=50000;

acc.perc=0.001; #0.1%

up.ind= nb_simul.val*acc.perc; ## 50 (top 0.1% among 50000);

# Matrix of simulated mu (mustar) and sigma (sigstar) values

par.mat=matrix(NA,ncol=2,nrow=nb_simul.val)

# Distance between summary statistics (xmin, xmed, xmax) and simulated values (ss1, ss2, ss3)

dist=rep(NA,nb_simul.val)

for (i in 1:nb_simul.val){

mustar=runif(1,xmin,xmax);

sigstar=runif(1,0,50)

# Generate estimates from pseudo data under Normal distribution

temp.sam=rnorm(n,mustar,sigstar)

ss1=min(temp.sam);ss2=median(temp.sam);ss3=max(temp.sam);

dist[i]=sqrt(sum((c(xmin,xmed,xmax)-c(ss1,ss2,ss3))^2))

par.mat[i,1]=mustar;

par.mat[i,2]=sigstar;

}

# Find top 0.1% shortest distance among 50,000 values of mu and sigma;

ind=sort(dist,index.return=T)$ix

output=par.mat[ind[1:up.ind],]

# Average of top 0.1% ABC estimates;

est= apply(output,2,'mean')

print(est) ;

# ABC estimates mean= 51.34 and SD = 16.46;

# Recall true Mean= 50.29 & SD= 16.47;
